# Calendar

**DOI:** 10.1155/S1463924682000492

**Published:** 1982


					Journal of Automatic Chemistry, Volume 4, Number 4 (October-December 1982), pages 195-196
Calendar
Editor's note
Organizers of conferences, seminars, etc.
should send details for inclusion in the
calendar as soon as the relevant infor-
mation is available and not later than three
months before the event.
November 1982
The Selection and Use of Liquid/Solid
Separation Equipment
One-day symposium with a commercial
exhibition on 24 November at UMIST,
Manchester, UK.
Details from Mr A. L. Clarke, Diamond
Shamrock (UK) Ltd, PO Box 1, Eccles,
Manchester MJO OBH, UK.
December 1982
Teaching Microcomputing--Taking
Stock of the Situation
Joint meeting of the Automatic Methods
Group and the Education and Training
Group ofthe Royal Society ofChemistry.
December at the Laboratory of the
Government Chemist, London.
Contact Dr C. J. Jackson, Occupational
Hygiene Laboratory, 403 Edgware Road,
London NW2 6LN.
Automated Automotive Analysis
To be held on 2 December at the BP
Research Centre, Sunbury-on-Thames,
UK.
Contact Dr C. J. Jackson, Occupational
Hygiene Laboratory, 403 Ed.gware Road,
London NW2 6LN.
Mesucora 82 and Physique 82
To be held from 6-11 December at Porte
de Versailles, Paris.
Further information from SEPIC/
Mesucora-Physique, 40 rue du Colisbe,
75381 Paris Cedex 08.
Seminar on Optical Sensing: Techniques,
Benefits and Costs
Organized by the National Physical
Laboratory and Sira, and sponsored by
the Institute of Measurement and
Control and the Institute of Electrical
Engineers. To be held from 7 to 8
December at the NPL in Teddington,
UK.
Contact the Commercial Department,
Sira Institute Ltd, South Hill, Chislehurst,
Kent BR7 5EH, UK.
Royal Society of Chemistry--Analytical
Division (Special Techniques Group):
Symposium on Unusual and Difficult
Samples
To be held on 8 December at Imperial
College, London.
Further informationfrom Dr J. G. Williams,
The Laboratory of the Government
Chemist, Stamford Street, London
SE1 9NQ.
Flow-Injection Analysis
Meeting organized by the Royal Society
of Chemistry's Analytical Division--
North East Region. To be held on 8
December at the City Polytechnic,
Sheffield, UK.
Details from C. L. Denton, 20 Bedford
Road, Nunthorpe, Middlesborough,
Cleveland, UK.
Symposium on 'Surface Analysis'
To be held on 15 and 16 December at
St. Catherine's College, University of
Oxford, Oxford, UK.
Contact Miss P. E. Hutchinson, Analytical
Division, Royal Society of Chemistry,
Burlington House, London W1 VOBN.
Course on Fibre Optics for
Instrumentation
Organized by UMIST and the Sira
Institute. To be held from 15 to 17
December at UMIST, Manchester, UK.
The course will also be run from 28 to 30
June 1983.
Contact the Conference Unit, Sira
Institute Ltd, South Hill, Chislehurst,
Kent BR7 5EH, UK.
February 1983
Applications of Automated Atomic
Spectroscopy to Environmental Analysis
Joint meeting of the Royal Society of
Chemistry's Automatic Methods Group,
South East Region and Atomic
Spectroscopy Group. To be held at the
Occupational Hygiene Laboratory,
London, on 16 February.
Contact Dr C. J. Jackson, Occupational
Hygiene Laboratory, 403 Edgware Road,
London NW2 6LN.
Seminar on Ion-Selective Electrodes and
Related Sensors: Recent Industrial
Applications
To be held on 22 February at the Sira
Institute, Chislehurst, UK.
Further information from the Conference
Unit, Sira Institute Ltd, South Hill,
Chislehurst, Kent BR7 5EH.
March 1983
34th Pittsburgh Conference and
Exposition on Analytical Chemistry and
Applied Spectroscopy
To be held from 7-12 March in Atlantic
City, New Jersey, USA.
Authors should contact Mrs Linda Briggs,
Program Secretary, Pittsburgh Conference,
437 Donald Road, Department J-212,
Pittsburgh, Pennsylvania 15235, USA; and
exhibitors Mr Paul E. Bauer, Exposition
Chairman, at the same address.
Problems in Process Analysis
Royal Society of Chemistry's Automatic
Methods Group and the Industrial
Analytical Instrumentation Group ofthe
Institute of Measurement and Control.
To be held on 8 March at the Bloomsbury
Centre Hotel, London.
Contact Dr C. J. Jackson, Occupational
Hygiene Laboratory, 403 Edgware Road,
London NW2 6LN.
Microcomputers in Analytical Chemistry
Northern Ireland Region: Royal Society
ofChemistry. To be held on 15 March at
Queen's University, Belfast, UK.
Contact W. J. Swindall, Department of
Chemistry, David Keir Building, Queen's
University, Belfast BT9 SAG, UK.
April 1983
International Symposium on Electro-
analysis in Biomedical, Environmental and
Industrial Sciences
Organized by the Electroanalytical
Group and Western Region of the
Analytical Division of the Royal Society
of Chemistry. To be held from 5-8 April
at UWIST, Cardiff, UK.
Contact the Short Courses Section,
UWIST, King Edward VII Avenue,
CardiffCF1 3NU, UK.
Royal Society of Chemistry Annual
Chemical Congress
To be held from 11 to 13 April at the
University of Lancaster, UK.
Details from Dr John F. Gibson, Royal
Society of Chemistry, Burlington House,
London W1 VOBN.
May 1983
VIIth International Symposium on
Column Liquid Chromatography
To be held from 2 to 6 May in Frankfurt,
FR Germany.
Details from Dr J. Wendenburg, c/o
Gesellschaft Deutscher Chemiker,
Varrentrappstrasse 40-42, D 6000
Frankfurt 90, FR Germany.
195
Calendar
June 1983
Tectronica 83
To be held at Earls Court in London from
14-17 June 1983.
Contact Industrial & Trade Fairs Ltd,
Radcliffe House, Blenheim Court, Solihull,
West Midlands B91 2BG, UK.
Modern Methods of Food Analysis
A basic science symposium organized by
the Institute of Foods Technologists. To
be held from 17-18 June in New Orleans,
USA.
Contact either Dr John Whitaker,
University ofCalifornia, Davis, California
95616, USA; or Dr Kent K. Stewart,
Department ofFood Science & Technology,
Virginia Polytechnic Institute and State
University, Blacksburg, Virginia 24061,
USA.
ISA at Transducer/Tempcon 83
The Instrument Society of America is
organizing a two-day event at the
Transducer/Tempcon exhibition and
conference at the Wembley Conference
Centre, London, from 28-30 June. The
event will be subject to separate
registration.
Informationfrom Norma Thewlis, Trident
International Exhibitions Ltd, 21
Plymouth Road, Tavistock, Devon PL19
8AU, UK.
July 1983
Computers in Automation and Laboratory
Management
The fifth Summer School of Automatic
Chemical Analysis. The course is
residential and organized by Professor
J. Betteridge, D. Porter and Dr P.
Stockwell. To be held from 10 to 15 July
at the University of Sussex, UK.
Details from Beverly Humphrey, 176a
North View Road, London N8 7NB.
SAC 83: International Conference and
Exhibition on Analytical Chemistry
Organized by the Analytical Division of
the Royal Society of Chemistry. To be
held from 17-23 July at the University of
Edinburgh.
Contact Miss P. E. Hutchinson, Analytical
Division, Royal Society of Chemistry,
Burlington House, London W1 VOBN.
November 1984
14th Annual Symposium on the Analytical
Chemistry of Pollutants and the 3rd
International Congress on Analytical
Techniques in Environmental Chemistry
To be held from 22-24 November 1984 at
the Palacio de Congresos in Barcelona,
Spain.
For further information contact 14th
Annual Symposium--3rd International
Congress--Expoquimia, Avda. Reina M.
Christina, Barcelona 4, Spain.
COURSE ANNOUNCEMENT
UMIST/Sira practical course on fibre optics for instrumentation: basics, sensors and systems
To meet continuing demand, the UMIST/Sira course on fibre optics for instrumentation will be repeated on 15-17 December
1982 and again on 28-30 June 1983 at UMIST, Manchester, UK. This three-day training course consists of lectures,
demonstrations, practical sessions and discussion groups. The aim is to provide participants with a basic knowledge offibre
optics and an appreciation of the technical and commercial potential of fibre optics as applied to instrumentation.
The course is intended for potential users offibre optics in conjunction with instrumentation. Participants need not have a
detailed prior knowledge of fibre optics.
The lectures, in conjunction with the demonstrations and practical exercises, cover the following topics:
Basic optics and principles of fibre optics
Optical radiation sources and detectors
Optical effects of potential use in measurement
Fibre optic cables
Couplers, connectors and splices
Optical sensors, with case studies
Systems design
Fibre optic connectors between instruments
Economics of fibre optic systems
The future for fibre optics in instrumentation.
The course costs 345, which includes three days' accommodation and meals. Detailsfrom Sira's Conference Unit at South Hill,
Chislehurst, Kent BR7 5EH, UK. Tel. O1 467 2636.
196

				

